# Effect of Red Orange Juice Consumption on Body Composition and Nutritional Status in Overweight/Obese Female: A Pilot Study

**DOI:** 10.1155/2017/1672567

**Published:** 2017-03-20

**Authors:** E. Azzini, E. Venneria, D. Ciarapica, M. S. Foddai, F. Intorre, M. Zaccaria, F. Maiani, L. Palomba, L. Barnaba, C. Tubili, G. Maiani, A. Polito

**Affiliations:** ^1^Council for Agricultural Research and Economics (CREA), Research Center for Food and Nutrition, Via Ardeatina 546, 00178 Rome, Italy; ^2^Diabetes Unit, San Camilo-Forlanini Hospital, Rome, Italy

## Abstract

The main objective of this research was to determine whether a commercial orange juice rich in anthocyanins could have an effect on body weight and on clinical parameters related to obesity including antioxidant status, lipid profile, and metabolic and inflammatory biomarkers. 11 women with an average BMI of 34.4 ± 4.8 kg/m^2^ were enrolled in a pilot study. Over a period of 12 weeks they received 500 mL daily dose into two doses (250 mL) of commercial red orange juice (COJ). The biochemical parameters were measured at baseline and at the end of the study (12 weeks). One month later upon free diet, a follow-up was performed measuring the same variables. The daily consumption of 500 mL of COJ had no significant effects on body weight, while there was a decrease in total cholesterol and LDL cholesterol. The grade of obesity implies different changes in inflammation biomarkers. In obese women, our data do not seem to support evidence that commercial red orange juice consumption acts as functional food preventing obesity and metabolic disorders such as insulin resistance and/or inflammatory status.

## 1. Introduction

Obesity is well recognized as one of the major public health issues worldwide and its prevalence is increasing [1, 2]. Overweight and/or obesity rates in Italy, like other Western countries, are estimated to be 32% and/or 10%, respectively [3]. Obesity with fat accumulation in the abdominal area, especially in the visceral compartment, may be associated with a higher prevalence of diabetes mellitus, hypertension, dyslipidemia, and coronary heart disease than gluteal-femoral obesity. The specific etiopathogenetic mechanism is not clear, although some evidence suggests that, as a major endocrine and secretory organ, visceral adipose tissue could play a key role in these metabolic complications releasing several hormones, cytokines, and other vasoactive substances [4, 5].

Obesity is commonly caused by eating excessive calories more than energy requirement needs for a long time. As widely known food intake and energy expenditure are regulated by complex interactions between the individual's genetic background and environmental factors [6].

In humans, it is estimated that genes are responsible for only 40% in determining body mass changes while the increased prevalence of obesity in the last 20 years is mainly due to environmental factors in determining high energy intake and low energy expenditure. Lifestyles have become increasingly more sedentary leading to positive energy balance and weight gain.

Recently it was shown that preventing some metabolic disorders associated with obesity, such as diabetes mellitus, was possible by diet and lifestyle modification [7, 8].

In particular, several epidemiological studies confirmed an inverse association between higher fruit and vegetable consumption and the occurrence of cardiovascular disease, cancer, and chronic-degenerative disease [9–13]. Fruits and vegetables supply vitamins, minerals, trace elements, and dietary fiber and contain a unique combination and amount of compounds called nutraceuticals or phytochemicals with surprising beneficial properties (nutritional and pharmacological). Various phytonutraceuticals with antioxidant properties are able to counteract the inflammatory processes, to delay aging processes and to slow cellular degeneration.

Recently, of great interest is the dietary consumption of the anthocyanins, a water-soluble pigments belonging to flavonoids class. Their most recognized properties are to confer a wide range of colors with red, blue, purple, and violet in many fruits and vegetables and to exert potential health benefits in the free-radical scavenging, antioxidant capacities and antibacterial activity [14–16].

Accumulated research evidence has also highlighted the anthocyanins' role in rats supplemented by anthocyanin-rich diet, in the improvement of blood lipids, ameliorated inflammation, greater decrease in body weight, and better weigh control [17–19].

Few studies were designed to verify the lipid-lowering and/or antioxidative capacity as well as blood glucose and blood pressure control by anthocyanins intake in humans [20–22], while how these bioactive components could be implicated in regulating body weight is not completely clear.

In view of the foregoing considerations, to improve the knowledge and to highlight the health-promoting properties of anthocyanin rich foods, the effect of red orange juice consumption on nutritional anthropometry and on nutritional/metabolic status (blood lipids, vitamin, and hormonal and antioxidant status) in overweight/obese women was studied in a pilot study.

## 2. Materials and Methods

### 2.1. Red Orange Juice

A commercial pasteurized red orange juice (COJ) produced by Ortogel (Catania, Italy) was used as the anthocyanins source.  Table 1 shows the energy and nutrients provided by 100 mL of COJ as reported by Ortogel. Frozen blood orange juice was given every 15 days to each participant and stored at 4°C before consumption.

### 2.2. Subjects

The study was conducted in accordance with the Declaration of Helsinki on the human trial performance and informed consent was provided by participants. They were informed about the research purpose and procedure, benefits, and risks, having the freedom to drop out from the study at any time. The study protocol was approved by the Ethical Committee of the San Camillo Forlanini Hospital in Rome. In this study, the mean age of the enrolled women was 36 ± 7 years, while an average BMI of 34.4 ± 4.8 kg/m^2^ indicates a presence of obesity. Overweight and/or obese women were recruited from San Camillo Forlanini Hospital and primary care physicians. Volunteers were screened considering the following exclusion criteria: presence of pathological conditions representing a risk for volunteers or likely to influence the outcomes, use of vitamin and/or mineral supplements or drugs in the last 3 months prior to the study, unusual dietary habits (vegetarians, vegans), regular consumption of alcohol > 20 g/day, and smoking habits. Information concerning medical and surgical history and use of drugs or nutritional supplements was collected by a general interview, as well as data on smoking and alcohol consumption. Anthropometric measurements (height and body weight) were performed in the morning in fasting conditions according to the standardized procedure [23]. After this screening, 20 adult women were enrolled, six volunteers declined to participate, three of them dropped out of the study, and the final sample was of 11 subjects.

### 2.3. Study Design

The 12-week study was a controlled feeding trial conducted by daily intake of blood orange juice.  Table 2 summarizes the experimental design. According to literature data and on the basis of juice composition, enrolled subjects drunk 500 mL daily dose (two doses of 250 mL) before mealtime, corresponding to 250 mg of anthocyanins/day. Systolic and diastolic blood pressure and anthropometric measurements were performed before, every four weeks over intervention phase and at the end of COJ consumption. Biochemical parameters were measured at baseline and at the end of the study including the assessment of blood lipids and vitamin and hormonal and antioxidant status. One month later upon free diet, a follow-up was conducted on the same variables. Furthermore, subjects were asked to maintain their usual physical activity, lifestyle, and diet. Monitoring of diet was periodically carried out by 4 days of dietary records collected every 15 days; food intake data were converted into nutrient intake using the Italian food composition tables [24]. Information concerning medical conditions, use of drugs, or nutritional supplements was collected by a general interview, as well as data on lifestyle including smoking and alcohol consumption.

### 2.4. Methodologies

Anthropometric measurements including body weight, stature, circumferences, and skinfold thicknesses were assessed in accordance with the techniques described by Lohman et al. (1988).

Systolic and diastolic blood pressure was measured in mm Hg using Omron M6 (HEM-7001-E), after subjects had been resting for approximately 8–10 minutes.

Blood samples were collected in EDTA or heparin containing tubes. After centrifuging at 3000 rpm in refrigerate conditions the plasma was obtained and then stored at −80°C until analyses. Aliquots of plasma were used as shown below. Plasma Total Antioxidant Capacity was measured using Ferric Reducing Antioxidant Power (FRAP) method [25]; plasma Vitamins A and E determinations were carried out by HPLC as previously described by Maiani et al. [26]. Total ascorbic acid was extracted using the method described by Margolis and Schapira [27] and the quantitative analysis was performed using an HPLC system equipped with a coulometric detector (ESA model 580; Chelmsford, MA, USA) [28]. Total cholesterol, HDL cholesterol, triglycerides, and uric acid concentrations were measured using enzymatic tests (Sentinel Diagnostics, Milano, Italy). LDL cholesterol was estimated by the Friedewald's formula. CRP, TNF-alpha, leptin, adiponectin, and insulin levels were measured by ELISA using commercial kits (DRG International, Inc., BD Biosciences ELISA reagent and Boster Biological Technology, Ltd.). The homeostasis model assessment-estimated insulin resistance (HOMA-IR) was used to assess insulin resistance. HOMA-IR was calculated multiplying fasting plasma insulin (mU/L) by fasting plasma glucose (mg/dL) and then dividing by the constant 405 [29].

### 2.5. Statistics

The results are presented as means with their standard error. Differences between the end and the start of COJ consumption are given as means and their 95% confidence intervals (95% CIs). All data were tested for normal distribution using the Kolmogorov-Smirnov test. Difference between two phases on same variable was analysed by Paired *t*-test. Multiple-comparison post hoc correction by Bonferroni was applied. Nonnormal data were analyzed using nonparametric Kruskal-Wallis test. Results were considered significant at *P* < 0.05.

## 3. Results

**Figure 1:**
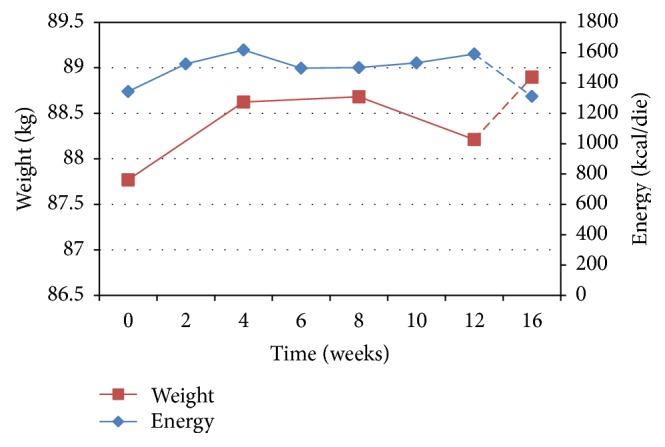
Mean changes in body weight over supplementation phase (12 weeks) and follow-up (one month later) by energy intake (mean ± SE).

No significant changes were observed in body weight on daily consumption of 500 mL of COJ at the end of a 12-week intervention (0.4 kg; 95% CI: −2.7 to 3.96) (Table 4).  Figure 1 shows the mean changes in body weight in association to energy intake. Although no significant differences in energy intake were present during the study, the small changes observed in body weight seem to follow the trends in energy intake. Furthermore, after COJ consumption, over one-month follow-up following free diet, an increase in body weight (about 1%), despite decreasing in energy intake, was observed.

Habitual energy, macronutrient, and micronutrient intake over a 12-week supplementation phase and one month later upon free diet (follow-up) are reported in Table 3.

According to dietary survey, no significant differences in habitual diet were observed. However, it should be taken into account that the orange juice consumption led to an increasing daily calories intake (+940 kJ/die), which inevitably affect the body weight, if a decreased energy intake or increased physical activity level are not implemented.

Table 5 displays some biochemical and hemodynamic parameters over COJ consumption (12 weeks) and one month later upon free diet (follow-up). COJ consumption determined a decrease corresponding in a mean value of −11 mg/dL (95% CI: −55 to 7) in total cholesterol levels and a significant decrease in LDL-cholesterol levels −9 mg/dL (95% CI: −30 to 12; *P* = 0.046). Moreover, over one month after COJ consumption (follow-up), moderate increases in total cholesterol (164 ± 14 versus 158 ± 13 mg/dL, resp.), triglycerides (123 ± 20 versus 106 ± 16 mg/dL, resp.), and uric acid levels (4.7 ± 0.4 versus 4.4 ± 0.4 mg/dL, resp.) were highlighted in addition to a significant increase (53.0 ± 8.0 versus 49.4 ± 7.5 *μ*g/dL, resp.; *P* = 0.04) in vitamin A and a decrease (1.24 ± 0.16* versus *1.28 ± 0.16 mg/dL, resp; *P* = 0.007) in vitamin E. Moreover a significant decrease in diastolic blood pressure (74 ± 2 versus 83 ± 3 mmHg, resp.; *P* = 0.01) was evidenced over follow-up. Measures of total ascorbic acid (1.18 ± 0.08 mg/dL), ascorbate (0.64 ± 0.12 mg/dL), and dehydroascorbate (0.53 ± 0.07 mg/dL) were higher after COJ consumption respect baseline (1.01 ± 0.05, 0.59 ± 0.07, and 0.42 ± 0.08 mg/dL, resp.).

Metabolic and inflammatory biomarkers over 12 weeks of COJ consumption and follow-up are presented in Table 6. A mean increase of 4.8 mg/dL (95% CI: −5.6 to 20.5) in glucose levels compared to baseline was present (105.7 ± 3.3 versus 100.9 ± 1.9 mg/dL, resp.). Furthermore, higher HOMA-IR of 0.6 (95% CI: −0.5 to 4.1) and higher leptin of 3.6 mg/dL (95% CI: −12.1 to 20.6) with lower adiponectin of −1.6 *μ*g/mL (95% CI: −8.5 to 4.3) levels were associated with consumption of COJ. In addition, an increased inflammatory response was present upon consumption of COJ.

**Figure 2:**
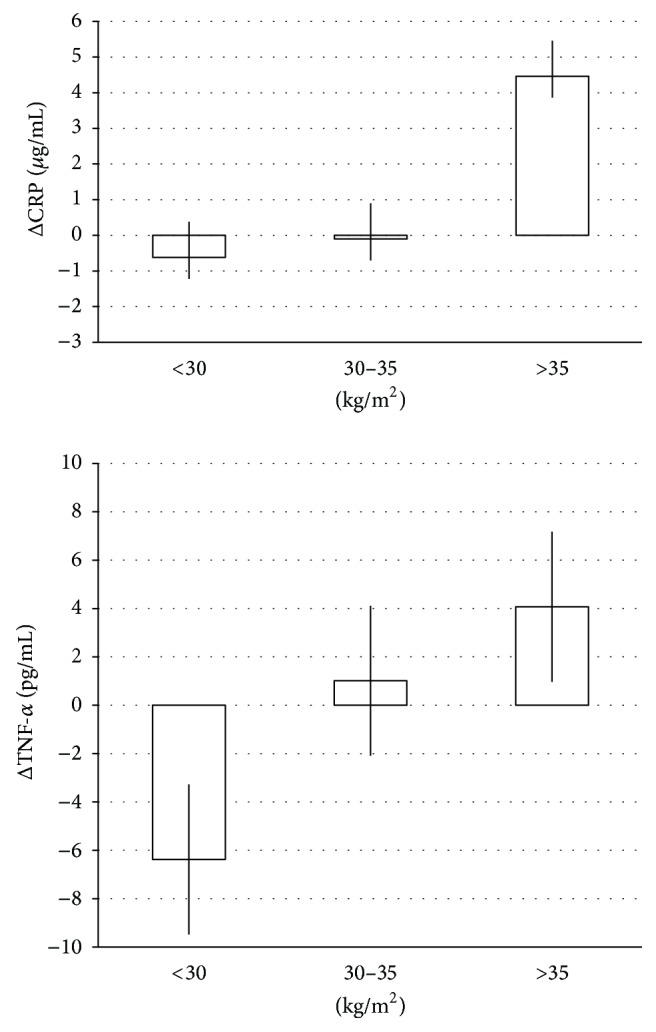
Pattern of change in CRP (*μ*g/mL) and TNF-*α* (pg/mL) by BMI levels.

**Figure 3:**
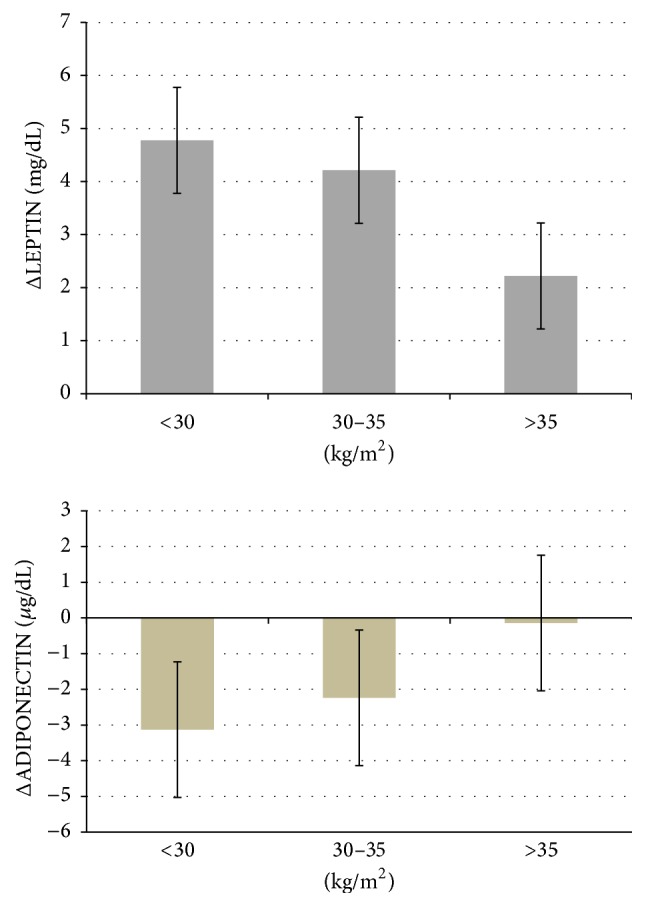
Pattern of change in leptin (mg/dL) and adiponectin (*μ*g/dL) by BMI levels.

Whereas no relationship was demonstrated between several parameters and BMI, to explore the possible influence of BMI on some variables, a further assessment was performed by distributing the results obtained at the end of supplementation by BMI classes. Indeed [30], as reported in Figure 2, a clear relationship between body mass classes (<30, 30–35, >35 kg/m^2^) and CRP increments with respect to baseline was relieved (−0.62 ± 0.62 *μ*g/mL, −0.10 ± 0.36 *μ*g/mL, and 4.46 ± 1.09 *μ*g/mL, resp.). Similar trend was present for TNF-*α* increments −6.38 ± 1.60 pg/mL, 1.01 ± 1.32 pg/mL, and 4.07 ± 1.48 pg/mL, respectively, by BMI classes. Moreover, Figure 3 reports the pattern of changes by BMI classes over 12-week COJ consumption in leptin and adiponectin levels. Subjects with the highest BMI classes showed a lower increase in leptin (37.4 ± 6.5 versus 35.2 ± 6.541; 34.4 ± 5.9 versus 30.2 ± 5.9; 18.3  ±  9.3 versus 13.6  ±  9.3, resp., for BMI <30, 30–35, and >35 kg/m^2^) as well as lower decrease in adiponectin levels.

## 4. Discussion

There is growing scientific interest in the nutraceuticals and functional foods and their potential protective effects on human health, both in prevention and in treatment of obesity and cardiovascular diseases. The orange juice contains a set of powerful bioactive molecules including flavonoids, carotenoids, vitamin C, folate, and other phytochemical compounds. It is also well known that COJ contains a high concentration of anthocyanins particularly cyanidin-3-glucoside, which seems to be implicated in the reduction of body weight and fat accumulation. The effect of COJ consumption on weight gain is controversial, especially because of its sugar content [31, 32]. In this study, COJ consumption resulted in augmented carbohydrates intakes with respect to baseline and one month later on free diet (follow-up). Experimental studies in animal models have shown a probable role of anthocyanins to attenuate obesity producing a decreased weight and adipose tissue and ameliorate insulin resistance [19, 33–35]. Previous studies on humans [36, 37] did not find significant anthropometric changes after regular orange juice consumption. Basile et al. [38] found a significant reduction in waist circumference in women over 8 weeks following COJ consumption but not in men. In addition, despite the higher fat intake among women, which could increase total cholesterol and LDL-cholesterol, there were important changes/improvement in their lipid profile. Same effect was reported by Dourado and Cesar [37]; an improvement of the lipid profile, evidenced by a reduction in total cholesterol and LDL-cholesterol in overweight subjects following COJ consumption, was observed, even if significant decreased lipids consumption was present. Total cholesterol and LDL-cholesterol levels decreased in our sample with slightly increase in dietary lipids, while triglycerides and HDL-cholesterol were unchanged. The absence in reducing triglyceride levels seems to confirm the primary effect due to gender, fasting glucose, insulin levels, insulin resistance, and total fructose intake [39]. In addition we failed to detect a raising HDL-cholesterol levels after COJ consumption was found in previous studies on humans [37, 38, 40]. Only two overweight subjects exhibited an increase of 3% with respect to HDL-cholesterol levels after COJ consumption (70 ± 4 versus 68 ± 4 mg/dL), while a mean reduction of 5% was present in subjects with a BMI > 30 kg/m^2^ (63.5 ± 3 versus 66.5 ± 3 kg/m^2^) (data not shown). These different outcomes may be explained by type of administration (fresh and/or commercial product or pure compound), dose, length of the study, and different sizes and clinical characteristics of the enrolled sample. In agreement with several studies, other components rather than or in addition to anthocyanins may contribute to enhancing lipid profile and to exert beneficial antiobesity effects [36, 41]. As it is well known, the obesity corresponds to a subclinical inflammatory condition that promotes the production of proinflammatory factors involved in the pathogenesis of insulin resistance [42]. Our sample at baseline was characterized by overproduction of proinflammatory mediators, such as circulating levels of C-reactive protein (4.45 ± 1.39 *μ*g/mL), TNF-*α* (33.24 ± 3.10 pg/mL), and insulin resistance (HOMA-IR 2.61 ± 1.27) and normal lipid profile, several of which are risk factors for cardiovascular diseases, metabolic syndrome, and diabetes. The only two overweight subjects showed a mean decrease of 34% and 23%, respectively, for CRP and TNF-*α* levels, while, among women with obesity (BMI > 30 kg/m^2^), mean increase of 39% and 5% was present, respectively. Thus, different grade of obesity implied different changes in inflammation biomarkers. The increase in blood glucose observed at 12 weeks after supplementation (105.7 ± 3.3 mg/dL) compared to baseline (100.9 ± 1.9 mg/dL) could be attributed to sugars contained in COJ (50 g/day), which may have influenced the plasma glucose levels as well as body weight (88.9  ±  4.9 versus 87.8  ±  4.5 kg). Factors associated with diet of subjects should be excluded because there were no significant changes throughout the study. In spite of increases of the ascorbate and dehydroascorbate levels, COJ consumption causes an increase of the DHA/AA ratio (83%) compared to baseline (72%). As reported [43] higher ratio may relate to the varied metabolic roles of the vitamin instead of inadequate dietary vitamin C intake. On the other hand, the decrease of uric acid and vitamin E seem to justify the decline in the levels of FRAP which contribute to the 60% and 5%, respectively [25].

Li et al. [44] have demonstrated that anthocyanin supplementation exerts beneficial metabolic effects in subjects with type 2 diabetes by improving dyslipidemia, enhancing antioxidant capacity, and preventing insulin resistance. Asgary et al. [45] have reported beneficial effects on the physiological characteristics of healthy volunteers particularly in some of inflammatory markers after the consumption of commercial and fresh orange juice. In our study, COJ consumption in obese women was associated with increased metabolic abnormalities associated with insulin resistance; only two overweight subjects showed a HOMA-IR value < 2.2 associated with a reduction of 14% after 12 weeks of COJ consumption. Obese women (>30 kg/m^2^) showed a mean increase of 39% in HOMA-IR value at the end of supplementation phase (4.2 ± 0.6 versus 3.5 ± 0.6). Higher plasma leptin concentrations (32.4 ± 4.1 versus 29.0 ± 4.4 mg/dL) and lower adiponectin levels (9.54 ± 1.33 versus 7.90 ± 1.08 *μ*g/dL) were highlighted suggesting increased obesity-related complications. In fact, proinflammatory mediators such as TNF-*α* and CRP slightly increased, as well as a decreased antioxidant status including FRAP, uric acid, vitamin A, and vitamin E which is evidenced. As reported by Fantuzzi [5], the mechanism by which the insulin-resistant state is associated with low levels of adiponectin is not clear while its anti-inflammatory role appears confirmed. Adiponectin reduces the production and activity of TNF-*α*; in contrast, in obese subjects increased TNF-*α* production could explain the decline of adiponectin levels. Hajri et al. [46] have reported that reduced insulin stimulation and increased TNF-*α* production are among the factors that contribute to the decline of adiponectin production and alteration of isomer composition in obese insulin-resistant subjects. Furthermore, some evidence indicates that TNF-*α* is an important player in the state of insulin resistance observed during obesity by interfering with insulin signaling [47]. Castro et al. [48] highlighted a probable pathophysiological and molecular mechanisms involved in the link between increased visceral adipose tissue, insulin resistance, and comorbidities. The mechanisms involved in the etiopathogenesis of insulin resistance related to obesity occur due to prereceptor, receptor, and/or postreceptor impairments, mainly to insulin receptor downregulation secondary to hyperinsulinemia (receptor) and inhibition of the intracellular cascades by several adiposity-related factors (e.g., impaired adipokines and/or cytokines secretion) (after receptor) [49]. At last, our data support the evidenced strong relationship between leptin and adiponectin as well as their modulation by BMI and dietary pattern diet; the linkage between leptin resistance and obesity was confirmed, too.

## 5. Conclusion

Although our research is a pilot study at low subjects stratifying by BMI classes and our findings cannot be generalized, they might be helpful in planning further studies design and supplementary knowledge.

In obese women our data do not seem to support evidence that COJ consumption acts as functional food and could be consumed as part of a healthy diet to prevent obesity and metabolic disorders such as insulin resistance and/or inflammatory status. We confirm the role of adipose tissue as endocrine and secretory organ in releasing adipokines and proinflammatory molecules. The grade of obesity implies different changes in inflammation biomarkers. Our findings support the hypothesis that COJ consumption was significantly associated with favorable effects on total cholesterol and LDL-cholesterol levels in subjects with normal lipid profile.

In the light of the aforementioned considerations and results we suggest the consumption of freshly squeezed orange juice, without added sugars in obese women as part of a controlled diet or alternatively taking into account the global energy diet. The combination with increasing physical activity for maintaining a healthy body weight through healthier food choices could improve the comorbidities related to the obesity.

## Figures and Tables

**Table 1:** Commercial orange juice: nutritional values per 100 mL^*∗*^.

Energetic value (kJ)	188
pH	3.6
Total soluble solids (°Brix)	11.2
Proteins (g)	0.6
Fats (g)	0.1
Carbohydrates (g)	10.0
Vitamin C (mg)	55
Anthocyanins (mg)	>50

^*∗*^Nutrients amount on data fixed by Ortogel.

**Table 2:** Study design.

	Time →							
	Baseline	2 weeks	4 weeks	6 weeks	8 weeks	10 weeks	12 weeks	Follow-up
Medical examination	x		x		x		x	x
Anthropometric measurements	x		x		x		x	x
Blood withdrawn	x						x	x
Lifestyle questionnaire	x							
Food diary	x	x	x	x	x	x	x	x

**Table 3:** Habitual energy, macronutrients, and micronutrient intake over supplementation phase (12 weeks) and one month later (follow-up).

	Before COJ	After COJ	Follow-up
Energy (kJ/die)	5623 ± 370	5704 ± 37	5489 ± 426
Proteins (g/die)	64 ± 4	55 ± 3	56 ± 2
Lipids (g/die)	58 ± 4	56 ± 8	54 ± 5
PUFA (g/die)	5.8 ± 1.6	5.2 ± 1.9	4.9 ± 1.0
MUFA (g/die)	8.7 ± 1.2	9.2 ± 2.0	8.5 ± 1.0
SFA (g/die)	14.7 ± 1.2	14.8 ± 1.7	12.8 ± 1.4
Carbohydrates (g/die)	145 ± 17	165 ± 11	154 ± 14
Anthocyanins (mg/die)	52 ± 8	41 ± 5	35 ± 6
Ascorbic acid (mg/die)	78 ± 17	62 ± 11	67 ± 8
Fibra	11.6 ± 1.3	11.0 ± 1.0	11.9 ± 0.4
Iron (mg/die)	6.75 ± 0.49	6.53 ± 1.47	6.82 ± 1.40
Calcium (mg/die)	406 ± 41	324 ± 50	422 ± 46
Retinol eq. (*μ*g/die)	714 ± 101	686 ± 114	916 ± 201
*α*-Tocopherol (mg/die)	8.51 ± 0.88	8.94 ± 1.15	9.12 ± 0.87

Data are expressed as mean ± SE.

No statistical difference between phases.

**Table 4:** Changes in body composition following consumption of 500 mL of COJ.

	Before COJ	After COJ	*P*	Δ^a^ (95% CI)
Body weight (kg)	87.8 ± 4.5	88.9 ± 4.5	n.s.	0.4 (−2.7 to 3.96)
BMI (kg/m^2^)	34.4 ± 1.4	34.6 ± 1.5	n.s.	0.2 (−1.2 to 1.7)
Waist circumference (cm)	96.0 ± 2.9	96.0 ± 3.0	n.s.	−0.0 (−5.6 to 6.3)
Hip circumference (cm)	119.38 ± 3.0	119.06 ± 3.16	n.s.	−0.3 (−4.2 to 3.0)
WHR	0.807 ± 0.02	0.808 ± 0.02	n.s.	0.007 (−0.011 to 0.06)

Data are expressed as mean ± SEM; n.s., not significant.

ANOVA: after COJ versus before COJ.

Δ^a^: differences from after to before COJ (mean values and their 95% confidence intervals).

WHR: waist/hip circumferences.

**Table 5:** Biochemical and hemodynamic parameters over 12 weeks following COJ consumption and one month later on free diet (follow-up).

	Before COJ	After COJ	*P*	Δ^a^ (95% CI)	Follow up	*P*
Systolic pressure (mmHg)	125 ± 5	131 ± 5	n.s.	6 (−15 to 16)	122 ± 4	n.s.
Diastolic Pressure (mmHg)	81 ± 2	83 ± 3	n.s.	3 (−10 to 10)	74 ± 2	0.01
FRAP (*μ*molFe^2+^/L)	832 ± 45	827 ± 42	n.s.	−5 (−93 to 116)	841 ± 61	n.s.
Uric acid (mg/dL)	4.6 ± 0.4	4.4 ± 0.4	n.s.	−0.2 (−0.9 to 1.0)	4.7 ± 0.4	n.s.
Total cholesterol (mg/dL)	170 ± 14	158 ± 13	n.s.	−11 (−55 to 7)	164 ± 14	n.s.
Triglycerides (mg/dL)	104 ± 21	106 ± 16	n.s.	3 (−108 to 89)	123 ± 20	n.s.
HDL-cholesterol (mg/dL)	66 ± 3	63 ± 2	n.s.	−3 (−15 to 4)	65 ± 2	n.s.
LDL-cholesterol (mg/dL)	83 ± 12	74 ± 13	0.046	−9 (−30 to 12)	74 ± 13	n.s.
Vitamin A (*μ*g/dL)	52.1 ± 8.3	49.4 ± 7.5	n.s.	−2.7 (−22.5 to 7.6)	53.0 ± 8.0	0.04
Vitamin E (mg/dL)	1.32 ± 0.16	1.28 ± 0.16	n.s.	0.08 (−0.40 to 0.33)	1.24 ± 0.16	0.007
Vitamin C (mg/dL)	1.01 ± 0.05	1.18 ± 0.08	n.s.	0.17 (−0.16 to 0.62)	1.06 ± 0.09	n.s.
AA (mg/dL)	0.59 ± 0.07	0.64 ± 0.12	n.s.	0.06 (−0.42 to 0.69)	0.52 ± 0.07	n.s
DHA (mg/dL)	0.42 ± 0.08	0.53 ± 0.07	n.s.	0.115 (−0.52 to 0.44)	0.54 ± 0.07	n.s

Data are expressed as mean ± SEM; n.s., not significant.

AA: ascorbic acid; DHA: dehydroascorbic acid.

*P*: ANOVA.

Δ^a^: differences from after to before COJ (mean values and their 95% confidence intervals).

**Table 6:** Metabolic and inflammatory biomarkers over supplementation phase (12 weeks) and follow-up (one month later).

	Before COJ	After COJ	*P*	Δ^a^ (95% CI)	Follow-up	*P*
Leptin (mg/dL)	29.0 ± 4.4	32.6 ± 4.1	n.s.	3.6 (−12.1 to 20.6)	31.2 ± 4.2	n.s.
Adiponectin (*μ*g/mL)	9.54 ± 1.33	7.90 ± 1.08	n.s.	−1.6 (−8.5 to 4.3)	7.66 ± 1.06	n.s.
Glucose (mg/dL)	100.9 ± 1.9	105.7 ± 3.3	0.07	4.8 (−5.6 to 20.5)	105.7 ± 2.7	n.s.
Insulin (mU/L)	10.6 ± 1.7	12.2 ± 2.1	n.s.	1.7 (−2.3 to 15.2)	11.4 ± 1.6	n.s.
HOMA-IR	2.61 ± 1.27	3.16 ± 0.54	n.s	0.6 (−0.5 to 4.1)	3.00 ± 0.47	n.s.
CRP (*μ*g/mL)	4.45 ± 1.39	5.91 ± 1.85	n.s	1.5 (−1.2 to 6.5)	6.10 ± 1.73	n.s
TNF-*α* (pg/mL)	33.24 ± 3.10	33.69 ± 2.69	n.s	0.4 (−10.7 to 20.4)	37.58 ± 2.03	n.s

Data are expressed as mean ± SEM; n.s., not significant.

*P*: ANOVA.

Δ^a^: differences from after to before COJ (mean values and their 95% confidence intervals).
